# Effect of a Physical Activity Intervention on the Metabolic Syndrome in Pakistani Immigrant Men: A Randomized Controlled Trial

**DOI:** 10.1007/s10903-012-9586-6

**Published:** 2012-03-11

**Authors:** Eivind Andersen, Arne T. Høstmark, Sigmund A. Anderssen

**Affiliations:** Department of Sport Medicine, Norwegian School of Sport Sciences, Ullevaal Stadium, Box 4014, 0806 Oslo, Norway

**Keywords:** Metabolic syndrome, Immigrant men, Physical activity, Randomized controlled trial

## Abstract

Physical activity (PA) is thought to prevent the metabolic syndrome (MetS), which is prevalent among south Asian immigrants in Western countries. The purpose of this study was to explore whether increasing PA improves the MetS and associated components in a group of Pakistani immigrant men living in Norway. One- hundred and fifty physically inactive Pakistani immigrant men were randomized to either a control group (CG) or an intervention group (IG). The 5 months intervention focused on increasing PA level, which was assessed using accelerometer recordings. Total PA level (counts min^−1^) increased significantly more in the IG than in the CG. The mean difference between the two groups was 49 counts min^−1^, which translates into a 15% (95% CI = 8.7% to 21.2%; *P* = 0.01) greater increase in total PA level in the IG than in the CG. Serum insulin concentration and waist circumference decreased more in the IG compared with the CG. Other MetS related factors and the prevalence of the MetS did not differ between the groups after the intervention. A five- month intervention program can increase PA level and cardiorespiratory fitness, and reduce insulin concentration and waist circumference. However this intervention program may not lower the prevalence of the complete MetS in Pakistani immigrant men.

## Introduction

A high prevalence of the metabolic syndrome (MetS), a clustering of metabolic abnormalities that includes glucose intolerance, dyslipidaemia and hypertension, has been reported both among south Asians residing in the Indian subcontinent (Pakistan, India and Bangladesh) [1, 2] and south Asian immigrants living in Western countries [3]. The high prevalence is a cause for concern because the syndrome predicts the development of type 2 diabetes, cardiovascular diseases and all-cause mortality [4, 5]. Factors proposed to explain this high prevalence of the MetS in this population include genetics, poor nutrition and low levels of physical activity (PA) [6]. Other factors such as a higher prevalence of insulin resistance, which seems to develop at an earlier age than in Caucasians (Europeans or other lighter-skinned populations) [7], and greater deposition of abdominal fat could also explain the higher prevalence of the MetS [7]. PA reduces the prevalence of the MetS [8, 9] and beneficially influences individual components of the syndrome. PA is widely recognized as an important part of the first- line treatment of the MetS [10]. There is a lack of PA intervention studies concerning MetS in general and among immigrants in particular. Thus, the overarching goal of the study was to evaluate the effect of PA among immigrants with respect to MetS.

A few randomized controlled trials have reported on the effect of PA on the MetS. Anderssen et al. [8] found a 23.5% reduction in the number of participants having the MetS in the exercise group versus 11.5% reduction in the control group after a one-year exercise intervention. In the Finnish diabetes prevention it was found that those who increased moderate- to vigorous PA (MVPA) the most were more likely to show resolution of the MetS or were less likely to develop the MetS [9]. A longitudinal study by Holme et al. [11] found that baseline leisure-time PA level was a significant predictor of the MetS at the follow-up 28 years later. It has previously also been shown that increases in PA and cardiorespiratory fitness improve single risk factors included in the definition of MetS. The above-mentioned studies provide evidence of the beneficial effects of PA on the MetS and the single MetS components. However, most studies on the effect of PA on the MetS and single MetS components have been conducted on persons of American or European descent.

We found only one prospective study on the effect of PA on the MetS components in south Asians [12]. In this uncontrolled study of 40 immigrant Pakistani women with the MetS living in Australia, a 12 weeks diet and exercise program increased PA level and reduced BMI and plasma concentrations of glucose, insulin, total cholesterol and TG. However, the effect of PA alone was not analyzed. An association between PA and single MetS components has been reported in some cross-sectional studies of south Asians. A high number of pedometer steps are associated with lower waist circumference in Asian Indians living in New Zealand [13], and a moderate level of PA is associated with healthier levels of fasting glucose, glucose-2 h and TG in Asian Indian immigrants living in the USA [14]. In this latter study, PA was not associated with HDLc concentration or blood pressure. Hayes et al. [15] found an inverse correlation between PA and insulin-2 h concentration but not with BMI, waist circumference, SBP or glucose or HDLc concentrations in south Asians. A study in England on “white Europeans” and south Asians found a lower PA level in south Asians. Whereas improvements in BMI, waist circumference, and the concentrations of glucose-2 h, HDLc and TG were beneficially associated with PA levels in the “white Europeans”, only waist circumference and HDLc concentration were associated with PA levels in south Asians [16].

PA should have had the same effect on different metabolic variables in our study group as found in Caucasians, although this effect has not been studied thoroughly. It is possible that the lack of a significant reduction in the prevalence of the MetS reflect underlying ethnic differences in the physiological responses to PA. There is emerging evidence that black Americans gain fewer protective effects than Caucasians for a given difference in objectively measured cardiorespiratory fitness, and pedometer studies suggest that the associations between PA and adiposity are weaker in Japanese populations than Caucasians [17–19]. In contrast, evidence from the American Diabetes Prevention Program found that the risk of diabetes was similarly reduced across ethnic groups after a lifestyle intervention [20]. These previous findings suggest that there could be ethnicity-specific physiological differences in the response to PA. Such differences should be investigated further.

Even though there seems to be a lack of knowledge about the metabolic effects of PA in south Asians, a recent consensus paper advocates PA as an important part of the treatment and prevention of the MetS [21]. However, as mentioned above, there seems to be some physiological differences between Caucasians and south Asians, which may explain, for example, why the latter group seems to respond differently than Caucasians to some drug treatments [7]. The physiological response to PA might also differ between south Asians and Caucasians.

Using a participatory approach, we recently developed an intervention program with the goal of increasing the PA level in a group of Pakistani immigrant men living in Norway. We reported previously that this intervention approach increased the PA level and reduced both the postprandial insulin concentration and waist circumference (manuscript submitted for publication). This observation raised the question whether the complete MetS or single components of the MetS can be influenced by the increase in PA produced by the intervention. Based upon our previous results, the aim of this paper was to examine whether the increase in PA in this group of immigrant men was associated with beneficial effects on (1) the complete MetS, and (2) single MetS factors.

## Methods

The Physical Activity and Minority Health Study was a randomized controlled trial whose main goal was to increase the level of PA in a group of Pakistani immigrant men. The study protocol was approved by the Regional Committee for Medical Research Ethics (ref. no. S-07300b) and the Norwegian Social Science Data Services (ref. no. 17212/2/KS), and all study participants gave written informed consent.

### Formative Research: Physical Activity Influences

To better understand why many Pakistani immigrants are physically inactive and how to positively influence their PA behavior, we conducted two focus groups with representatives from the male Pakistani immigrant group (*n* = 10 in each group, age ranged from 25 to 60 years). Each focus group meeting lasted approximately 2 h. The aims of these group meetings were to explore self-efficacy, expectations, expectancies, preferences and barriers to PA among the Pakistani immigrant men. These discussions indicated that the men had very few physically active friends or family members, had little knowledge about non vigorous PA, the link between PA and health and staying regularly physically active, and identified many barriers to PA (e.g., managing time) and did not know if they were able to overcome them, and they did not see many benefits of being regularly physically active. Using these results and numerous studies showing successful changes to the PA behavior by the use of social cognitive theory (SCT) constructs [22], we decided to target three primary SCT concepts to promote PA change: self-efficacy (i.e., confidence to perform PA), outcome expectancies (i.e., expected benefits and costs of performing PA) and the social environment (i.e., social support for PA from family and friends, physically active role models). The secondary SCT components included the physical environment (opportunities to perform PA) and behavioral capability (knowledge and skill).

#### Participants

**Fig. 1:**
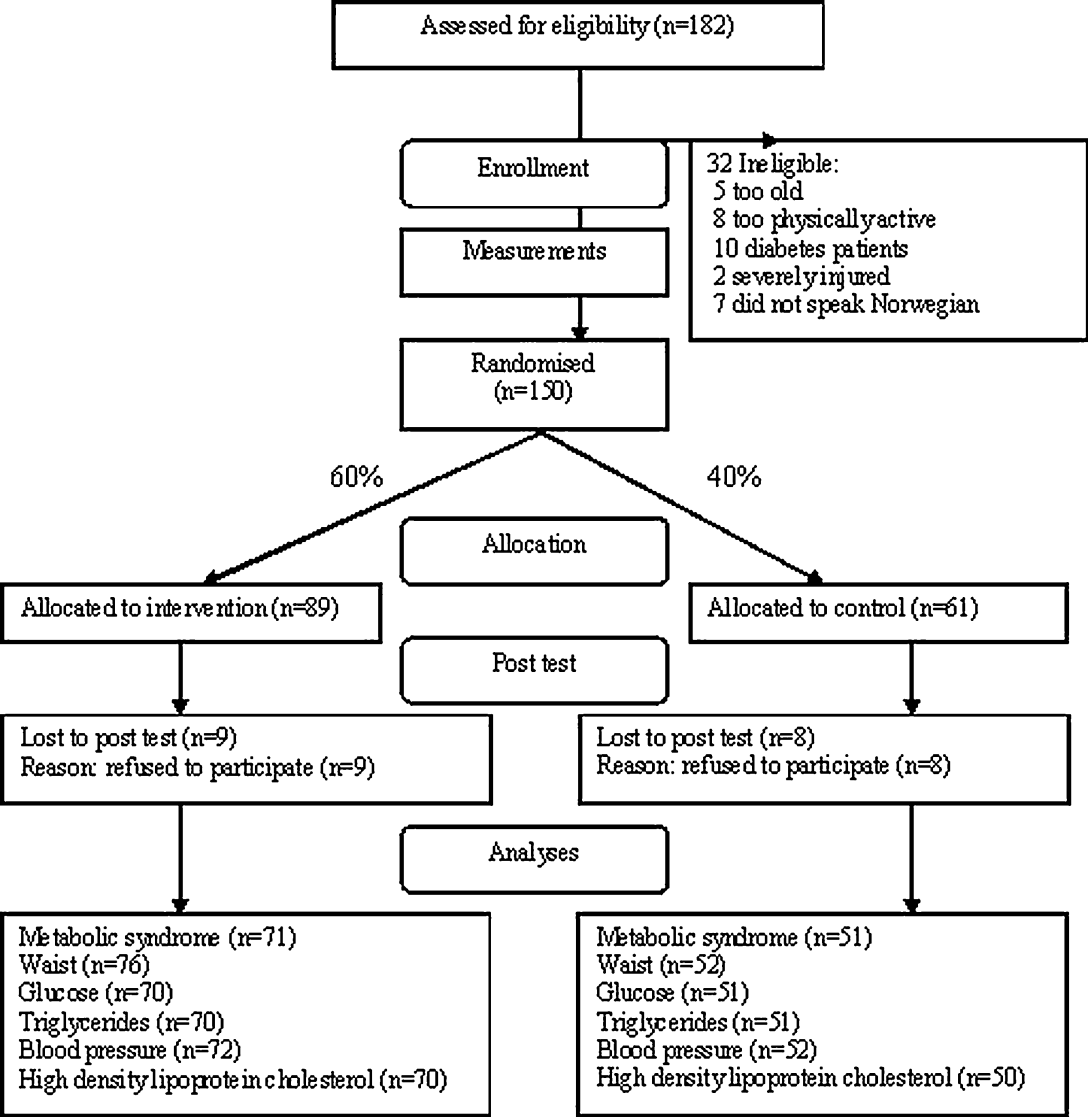
The flow of participants through the trial

Men living in Oslo, Norway, with a Pakistani background (either born in Pakistan or parents born in Pakistan) in the 25–60 year age group who were not physically active were eligible for the study (the definition of “not physically active” was exercising no more than twice a week at a moderate or high intensity for 30 min or more at a time or active commuting (e.g., cycling or walking to work on most days of the week)). Participants were recruited during the autumn of 2008. We gave a brief oral presentation about the project at six mosques and at various Muslim festivals in Oslo. One hundred and eighty-two men volunteered to participate in the study, 32 failed to meet the inclusion and exclusion criteria, giving 150 participants. Figure 1 presents the flow of participants through the trial.

### Intervention Program

In close collaboration with representatives from the target group, we developed an intervention to target the key SCT constructs via the following components: structured group exercise sessions twice a week led by an exercise physiologist, two group lectures, one individual counseling session, written material and a phone call. Table 1 provides an overview of how these components were conceptualized with reference to SCT. The intervention program lasted 5 months. The control group members received their baseline results approximately 2 weeks after the testing, and were offered organized exercise, one group lecture and written material following completion of the intervention period.

**Table 1:** Overview of the intervention components, attendance rates, behaviour change strategies and targeted social cognitive constructs

Intervention component	Dose	Description	Behaviour change strategy	Targeted construct
Structured group exercise	60 min twice a week	Participants could choose to attend one out of five different exercise facilities in Oslo. The different exercise groups were led by an exercise physiologist. The exercise training programme was designed as a low threshold activity. The sessions had the following structure: a 15 min warm-up with easy and fun games, 40 min of floorball and/or football plus some strength exercises and a 5 min cool down. Seven participants did not attend any of the sessions (one trained by himself and six were not motivated) and two were injured at the first exercise session. The mean attendance was 60% (range: 11–100%).	Provide opportunities for PA Increase social support for PA Promote mastery learning through skill training Improve knowledge and skill to perform PA Promote positive outcomes of PA Provide credible role models for PA	Environment Expectancies Self-efficacy
Group lectures	2 × 2 h	The lectures were conducted at the Norwegian School of Sports Sciences. The project leader led the classes. Major topics were: What is PA? PA and health link; short- and long term effects The harms of physical inactivity PA recommendations and how to achieve these Activity examples Setting small goals Identifying and reducing perceived barriers Making a PA plan Seeking social support Self reward Both attendees (95%) and non-attendees received written summaries of the lecturers.	Improve knowledge of PA options, including non-vigorous PA Improve knowledge on how to incorporate PA into the daily routine Enhance PA expectancies Improve goal setting for PA Improve problem solving of PA barriers Improve social support for PA	Social support Expectancies Self-efficacy
Individual counselling sessions	1 h	The counselling was based on the concept that all advice must match the participants’ experience of PA and degree of motivation. Together with the participant, the primary goal was to find activities that could be implemented in a usual week, with the sum of these activities enabling them to reach the PA recommendations. After discussing activity options, the participants set the goals they wanted to achieve over the five-month period. Finally, we discussed barriers by asking “What do you think can stop you from carrying out this activity plan?”, and the possible barriers, and solutions to them were discussed and written down. All participants completed this part of the intervention.	Identify opportunities for PA Improve knowledge and skill to perform PA Enhance goal setting for PA Promote mastery for PA Identify and problem solve barriers to PA	Social support Self-efficacy Expectancies
Phone call	5–15 min	T Three to five weeks before the first follow-up test, intervention participants in the intervention group were telephoned to discuss the activity plan, to make changes if necessary, and to encourage further efforts. All participants were reached within three attempts.	Provide feedback on PA behaviour Reinforce problem solving for PA Provide encouragement and help	Social support Self-efficacy

### Measurements

Each participant was examined for PA habits, cardiorespiratory fitness and MetS risk factors both before and after the five-month intervention. After an overnight fast, venous blood samples were drawn from an antecubital vein. Blood samples were centrifuged for 10 min at 2,500*g.* An oral glucose tolerance test was performed, in which 75 g of glucose in 200 ml of water was ingested and plasma glucose and insulin concentrations were measured before (fasting) and 2 h after (postprandial) ingestion of the glucose drink. A Modular P Machine (Roche, Japan) was used to measure the concentrations of HDLc (immunoturbidimetric assay), low density lipoprotein cholesterol (LDLc) (direct enzymatic method), triglycerides (TG) (enzymatic assay), glucose (photometric assay) and insulin (immunoassay). Waist circumference was measured in the standing position and after a light expiration horizontally to the chest, midway between the lower rib margin and the iliac crest. Weight was measured without shoes in light clothing using a SECA electronic scale (SECA model 767, Germany) to the nearest 0.5 kg. Height was measured without shoes with a transportable stadiometer (Harpenden; Holtain, Crymych, UK) to the nearest 0.5 cm. Body mass index (BMI) was calculated as weight divided by height squared (kg m^−^²). Blood pressure was measured automatically using an Omega non-invasive blood pressure monitor (In vivo Research, Inc., Orlando, FL., USA) in the morning after the participant had rested for 10 min in a quiet room. Three consecutive blood pressure measurements were performed with 1 min rest between each measurement. Blood pressure was recorded as the average value of the three recordings.

### The Metabolic Syndrome

The MetS was defined according to the criteria set by the International Diabetes Federation [10]. According to this definition, men must have central obesity, defined as waist circumference with ethnicity specific values (≥90 cm for south Asians) plus any two of the following four factors: serum TG concentration ≥1.7 mmol l^−1^, HDLc concentration ≤1.03 mmol l^−1^, systolic blood pressure (SBP) ≥130 mmHg or diastolic blood pressure (DBP) ≥85 mmHg or fasting plasma glucose concentration ≥5.6 mmol l^−1^.

By this definition, a person can have a maximum of five MetS components. If a MetS component was present, it was given the value 1 and 0 if not present. For example, a value of 3 would indicate three MetS-factors.

### Assessment of PA and Cardiorespiratory Fitness

Habitual PA was assessed with an Actigraph accelerometer (model 7164; ActiGraph, Fort Walton Beach, FL, USA). The participants were instructed to wear the accelerometer on the right hip during all waking hours for 7 days except while swimming and bathing. The epoch length was set to 1 min. When analyzing the accelerometer data, epoch periods with a value of 0 for 60 min (with allowance for two exceptions above 0) or longer were interpreted as “accelerometer not worn” and removed from the analyses [23, 24]. PA data were included if the participant had accumulated a minimum of 8 h of activity data per day for at least 2 days, regardless of the type of day (weekday or weekend). Accelerometer data were processed and analyzed using the SAS-based (version 9) (SAS Institute Inc. Cary, NC, USA) program CSA-Analyzer (http://csa.svenssonsport.dk). One hundred and forty-two participants had valid recordings at the baseline test (95%). Four lost their monitor and four had less than two valid days of recordings. At the post-test, 126 participants (84%) had valid recordings (intervention group *n* = 76, control group *n* = 50), 17 were lost to the post-test, five had less than 2 days of recordings, and two did not return their accelerometer.

Cardiorespiratory fitness was assessed by measuring oxygen consumption, which was defined as the highest measured oxygen consumption (VO_2_peak in ml kg^−1^ min^−1^). Oxygen consumption was measured during a maximum exercise test on a treadmill using a modified Balke protocol [25]. Gas exchange was sampled continuously into a mixing chamber every 30 s by having the participant breathe into a Hans Rudolph two-way breathing valve (2700 series, Hans Rudolph Inc., Kansas City, MO, USA) connected to a Jaeger Oxycon Pro gas analyser (Erich Jaeger GmbH, Hoechberg, Germany), which measured the oxygen and carbon dioxide content. The analyzer was volume- and gas calibrated before each test. The test result was approved when scoring ≥16 on the Borg 6–20-point rating of perceived exertion scale and when the respiratory quotient was >1.1. For safety reasons, we tested only those younger than 40 years (*n* = 99).

### Statistical Analyses

The mean and standard deviation (SD) were used to describe baseline data. Independent samples t tests were used to test differences between groups at baseline. The response to the intervention was measured as the difference between the corresponding final and baseline values for all variables (post-baseline; per protocol analysis without imputations). Repeated measures ANCOVA was used to test differences between the mean changes in the two groups, all analyses were adjusted for age. Effect size (ES) was calculated as: (changes in the control group ÷ changes in the intervention group)/SD in the control group. We analyzed all data using the Statistical Package for the Social Sciences (version 15, IBM, Inc., Chicago, IL, USA). There was a discrepancy between the inclusion criteria and the baseline PA levels. This is due to the use of different methods to assess PA; self-report was used for screening and an accelerometer was used in the testing. By any method, they would be characterized as having a low PA level [26].

## Results

### Drop-outs

Seventeen (11%) participants were lost to the post-test, nine in the intervention group and eight in the control group. The main reason given for not attending the post-test was a lack of interest. Except for a lower baseline PA level (counts min^−1^), the drop-outs did not differ on any variable from those who completed the program and testing.

### Baseline PA and Metabolic Characteristics

Table 2 displays the baseline characteristics for both the intervention and control groups. The prevalence of the MetS was high: 51% in the intervention group and 47% in the control group. The prevalence of the MetS and the components of the MetS did not differ significantly between the intervention and control groups. Overall, the most frequent contributing component to the MetS other than large waist circumference were low HDLc concentration (*n* = 79), high DBP and/or high SBP (*n* = 78) and high concentrations of TG (*n* = 69) and glucose (*n* = 40). Table 2 show that the men in both groups had a large waist circumference but that the levels of blood pressure and blood variables were within the normal range.

**Table 2:** Baseline descriptive characteristics of the intervention and control group

Characteristic	Intervention group (*n* = 89) Mean (SD)	Control group (*n* = 61) Mean (SD)	Mean difference (95% CI)
Age (years)	35.7 (6.1)	39.7 (9.2)	−3.9 (−6.6 to −1.2)*
MetS, *n* (%)^a^	46 (51)	29 (47)	
No. of MetS components	2.6 (1.1)	2.5 (1.2)	0.06 (−0.3 to 0.4)
Waist circumference (cm)	98 (9)	99 (11)	−1.1 (−4.6 to 2.3)
Triglycerides (mmol l^−1^)	1.9 (1.8)	2.0 (1.6)	−0.05 (−0.6 to 0.5)
HDLc (mmol l^−1^)	1.0 (0.2)	1.0 (0.2)	0.0 (−0.08 to 0.09)
LDLc (mmol l^−1^)	3.5 (0.6)	3.4 (0.9)	0.08 (−0.1 to 0.3)
Blood glucose (mmol l^−1^)	5.3 (0.7)	5.4 (1.1)	−0.1 (−0.5 to 0.1)
Systolic BP (mmHg)	119 (11)	119 (10)	−0.6 (−4.3 to 2.9)
Diastolic BP (mmHg)	85 (9.0)	85 (10)	0.2 (−2.9 to 3.4)

Data are presented as mean (SD) if not specified otherwise. *SD* standard deviation, *CI* confidence interval, *MetS* metabolic syndrome, *HDLc* high density lipoprotein cholesterol, *LDLc* low density lipoprotein cholesterol, *BP* blood pressure

^a^[10]

The intervention group had a higher total PA level (mean ± SD = 328 ± 138 counts min^−1^) than the control group (281 ± 118 counts min^−1^), but this was not significant after adjusting for age (40 counts min^−1^ mean difference, 95% CI = −5.6–85; *P* = 0.08). The amount MVPA was 35 ± 21 min day^−1^ for the intervention group and 28 ± 19 min day^−1^ for the control group. Furthermore, both the intervention and control groups spent many hours each day being physically inactive (8.5 ± 1.6 and 8.9 ± 1.5 h day^−1^ respectively) and had low mean VO_2_ peak values (33.9 ± 5.2 and 34.7 ± 6.5 ml kg^−1^ min^−1^, respectively). None of the PA variables or cardiorespiratory fitness differed between the two groups.

### Changes in Metabolic Characteristics and PA

**Fig. 2:**
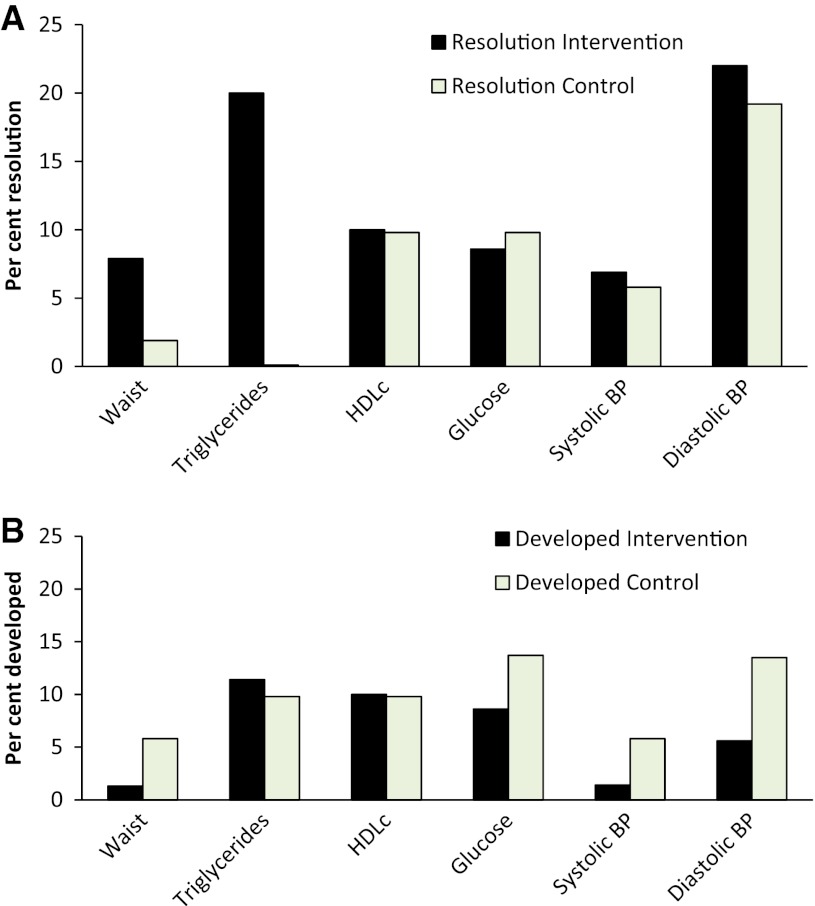
Incidence (%) of the resolution (**a**) and the development (**b**) of components of the metabolic syndrome during the intervention period for the intervention group (*black bars*) and the control group (*grey bars*). *HDLc* high density lipoprotein cholesterol. *BP* blood pressure

The prevalence of the MetS and resolution of the MetS did not differ between the intervention and control groups after the 5 months (Table 3). Information about the resolution and development of the MetS components are given in Fig. 2. Except for resolution of TG, no difference between the groups could be seen.

**Table 3:** Changes in the number of participants with the metabolic syndrome

		Intervention group	Control group	Total
Resolution of the MetS	Number	12	7	19
	Per cent	16.9%	13.7%	15.6%
Development of the MetS	Number	8	9	17
	Per cent	11.3%	17.6%	13.9%
No change	Number	51	35	86
	Per cent	71.8%	68.6%	70.5%
Total	Number	71	51	122
	Per cent	100.0%	100.0%	100.0%

Forty-six (51%) of the participants in the intervention group and 29 (47%) of the participants in the control group had the metabolic syndrome (MetS) at baseline. After the 5 months, 31 (34%) of the participants in the intervention group and 26 (43%) of the participants in the control group had the MetS

The waist circumference and insulin-2 h concentration were lower after the 5 months in the intervention group than in the control group. The two groups did not differ significantly on any other MetS-related components after the 5 months (Table 4).

**Table 4:** Response differences in the control and intervention groups

Characteristic	Intervention group (*n* range; 69–77) ∆ mean (SEM)	Control group (*n* range; 47–53) ∆ mean (SEM)	*F* value	*P* value*	Effect size	Adjusted mean df ± 95% CI
No. of MetS components	−0.2 (0.1)	−0.03 (0.1)	0.4	0.5	−0.08	−0.1 (−0.6 to 0.3)
Waist circumference (cm)	−1.9 (0.4)	1.7 (0.4)	25.2	<0.01	−1.06	−3.4 (−4.7 to −2.0)
Triglycerides (mmol l^−1^)	0.04 (0.1)	−0.02 (0.1)	0.4	0.5	0.09	0.1 (−0.2 to 0.4)
HDLc (mmol l^−1^)	0.00 (0.01)	−0.01 (0.01	0.1	0.7	0.08	0.008 (−0.03 to 0.05)
LDLc (mmol l^−1^)	−0.05(0.06)	0.02 (0.09)	1.1	0.2	−0.12	−0.07 (−0.3 to 0.1)
Glucose (mmol l^−1^)	−0.14 (0.05)	−0.06 (0.1)	0.7	0.3	−0.09	−0.1 (−0.4 to 0.1)
Systolic BP (mmHg)	−1.7 (0.9)	0.05 (1.3)	1.0	0.3	−0.17	−1.6 (−5 to 1.6)
Diastolic BP (mmHg)	−3.8 (0.8)	−0.9 (1.0)	3.1	0.08	−0.34	−2.5 (−5.3 to 0.3)

* adjusted for age. *SEM* standard error of the mean, *CI* confidence intervals, *MetS* metabolic syndrome, *HDLc* high density lipoprotein cholesterol, *LDLc* low density lipoprotein cholesterol, *BP* blood pressure

The mean difference in PA between the two groups after the intervention was 49 counts min^−1^, which translates into a 15% (95% CI = 8.7–21.2; *P* = 0.01) increase in total PA level in the intervention group compared with the control group. The time in MVPA increased by 6.4 min day^−1^ (95% CI = 0.5–12; *P* = 0.03) and VO_2_ peak by 3.6 ml kg^−1^ min^−1^ (95% CI = 1.8–5.4; *P* < 0.01) compared with the control group. Inactive time decreased in both groups and did not differ significantly between the groups after the 5 months.

## Discussion

PA level increased following this five-month intervention program, and this increase was accompanied by increased cardiorespiratory fitness and reduced serum insulin levels and waist circumference. However, the complete MetS did not change. To our knowledge, our study is the first randomized and controlled trial to investigate the effect of PA on the MetS in a group of south Asian immigrants.

The increases in PA level in the current study might be insufficient to correct the MetS factors and hence to reduce the prevalence of the MetS. The PA guidelines in many countries state that one must engage in 30–60 min of MVPA, preferentially on all days of the week, to avoid lifestyle-associated non-communicable diseases. The minutes of MVPA can be accumulated throughout the day but should be in episodes of >10 min. The average amount of MVPA in the current study was 35 min for the intervention group at baseline and this increased by 13 min after the 5 months intervention. However, this increase in PA did not induce any significant changes in single MetS components, perhaps because improvements were also observed in the control group. When only MVPA bouts of 10 min or more were counted, only a few of the participants were sufficiently physically active, suggesting that the intermittent nature of the PA pattern among the participants might not have provided sufficient health benefits that might have occurred with longer bouts of MVPA.

The PA guidelines are mainly based on studies of Caucasians, and the PA dose for improving health might vary depending on ethnicity. This question was addressed in the American Physical Activity Guidelines Advisory Committee report in 2008, which concluded that although they did not find any clinically significant differences between ethnic groups in the response to PA, too little evidence was available to draw firm conclusions [27]. The PA guidelines are based mainly on questionnaire data and it is possible that the guidelines will be revised once we have more objectively measured PA data on diverse populations. Future studies should investigate whether the current PA guidelines are applicable to south Asians or if the guidelines should be modified for this understudied population. Of note, we observed a significantly reduced waist circumference, a key variable in the MetS definition. Reducing weight is one of the most difficult changes to achieve in type 2 diabetes-prone people. We found no changes in sugar intake, but we did not control for changes in total energy intake, and therefore cannot rule out the possibility that those who increased their PA level also reduced their total energy intake.

It is also plausible that the lack of significant changes in the prevalence of the MetS, and its single components could be attributed to a too-short intervention period in this target group or that the potential to cause physiological changes was low; for example, blood pressure was well within the normal range at baseline.

There is some evidence that increasing PA can beneficially influence the concentration of high density lipoprotein cholesterol (HDLc) [28] and other lipids in south Asians [29]. These studies did not include a control group, and only a few cross-sectional studies have shown a correlation between PA and components of the MetS in south Asians [16, 30]. However, these studies have used self-reported measures of PA. Self-reported methods relies on the ability of the participants to recall and report PA and individuals may tend to over or under-estimate their levels of PA when they are asked to provide self-reported estimates. In addition, difficulties in demonstrating a relationship between self-reported PA and biological measurements have been reported [31]. These measurement errors are thought to be reduced by objective measurements, such as by using an accelerometer [32].

Adherence to the intervention was good. All the participants met for the individual counselling session, most of the men met for the group sessions (those who could not attend received written summaries of the lecture) and all were reached for the telephone follow-up. However, the attendance rate for the structured group exercises varied from 11% to 100% (mean 60%). Despite the large variability in the attendance rates in the exercise classes, we did not detect any associations between changes to the PA level or other measured variables and attendance rates.

Insulin is the main hormone regulating glucose metabolism, and improved insulin sensitivity could reduce the risk of diabetes. Insulin also plays an important role in regulating fat metabolism and blood pressure. The predominant underlying risk factors for the MetS appears to be insulin resistance, abdominal obesity, ageing and physical inactivity [33]. Among these, insulin resistance could be the essential cause of the syndrome [34]. The improved insulin sensitivity, reduced waist circumference, reduced sedentary time and increased PA level observed after the five-month intervention may, in time, reduce the prevalence of the MetS.

### Strengths and Limitations

Our study seems to be the first randomized controlled study to assess the effect of objectively measured PA on the MetS in a group of south Asians. Because there are no validated PA questionnaires for this group, the use of objective tools reduces the measurement error, although accelerometers also have some weaknesses (e.g., they underestimate the load of uphill walking and upper body movements). Like many other PA interventions, our study had some drop-outs, although the drop-out rate of 11% could be considered moderate in this kind of study and is unlikely to have influenced the results. However, since the drop-outs had a lower PA level we cannot rule out the possibility that the results are an underestimation of the true impact of the intervention. It is possible that the lack of significant changes in the MetS reflects the lack of statistical power and that including only persons with the MetS would have given different results.

## Conclusions

A five-month intervention program increased PA and cardiorespiratory fitness, and reduced serum insulin concentration and waist circumference, but did not reduce the prevalence of the MetS in Pakistani immigrant men living in Norway.
